# Anthropogenic Threats to Wild Cetacean Welfare and a Tool to Inform Policy in This Area

**DOI:** 10.3389/fvets.2020.00057

**Published:** 2020-02-28

**Authors:** Christine Nicol, Lars Bejder, Laura Green, Craig Johnson, Linda Keeling, Dawn Noren, Julie Van der Hoop, Mark Simmonds

**Affiliations:** ^1^Royal Veterinary College, Hatfield, United Kingdom; ^2^Marine Mammal Research Program, Hawaii Institute of Marine Biology, University of Hawaii at Manoa, Kaneohe, HI, United States; ^3^Centre for Sustainable Aquatic Ecosystems, Harry Butler Institute, Murdoch University, Perth, WA, Australia; ^4^Zoophysiology, Department of Bioscience, Aarhus University, Aarhus, Denmark; ^5^College of Life and Environmental Sciences, University of Birmingham, Birmingham, United Kingdom; ^6^Animal Welfare Science and Bioethics Centre, School of Veterinary Sciences, Tāwharau Ora, Massey University, Palmerston North, New Zealand; ^7^Department of Animal Environment and Health, Swedish University of Agricultural Sciences, Uppsala, Sweden; ^8^Conservation Biology Division, Northwest Fisheries Science Center, National Marine Fisheries Service, National Oceanic and Atmospheric Administration, Seattle, WA, United States; ^9^Department of Zoophysiology, Aaarhus University, Aarhus, Denmark; ^10^School of Veterinary Science, University of Bristol, Langford House, Langford, United Kingdom; ^11^HSI-UK, London, United Kingdom

**Keywords:** cetacean, animal welfare, anthropogenic threat, expert elicitation, Five Domains model

## Abstract

Human activities and anthropogenic environmental changes are having a profound effect on biodiversity and the sustainability and health of many populations and species of wild mammals. There has been less attention devoted to the impact of human activities on the welfare of individual wild mammals, although ethical reasoning suggests that the welfare of an individual is important regardless of species abundance or population health. There is growing interest in developing methodologies and frameworks that could be used to obtain an overview of anthropogenic threats to animal welfare. This paper shows the steps taken to develop a functional welfare assessment tool for wild cetaceans (WATWC) via an iterative process involving input from a wide range of experts and stakeholders. Animal welfare is a multidimensional concept, and the WATWC presented made use of the Five Domains model of animal welfare to ensure that all areas of potential welfare impact were considered. A pilot version of the tool was tested and then refined to improve functionality. We demonstrated that the refined version of the WATWC was useful to assess real-world impacts of human activity on Southern Resident killer whales. There was close within-scenario agreement between assessors as well as between-scenario differentiation of overall welfare impact. The current article discusses the challenges raised by assessing welfare in scenarios where objective data on cetacean behavioral and physiological responses are sparse and proposes that the WATWC approach has value in identifying important information gaps and in contributing to policy decisions relating to human impacts on whales, dolphins, and porpoises.

## Introduction

Human activities and anthropogenic environmental changes can have serious adverse effects on mammalian population abundance, biodiversity, and the environment. These concerns have for many years been addressed by conservation policies and strategies developed by national governments and local, national, and multinational non-governmental organizations (NGOs). Conservation has also been the focus of extensive research effort. Some human activities can separately or additionally cause great harm to the welfare of individual wild animals (1, 2). However, individual animal welfare is often overlooked, and the fundamental concept of welfare is absent in most international wildlife and conservation laws and practices (3). Only recently has ethical and scientific work on wild animal welfare increased, accompanied by growing interest from NGOs and other international organizations in developing policies that meet conservation goals while recognizing the ethical dimension of actions that affect individuals. The draft proposal for a Universal Declaration on Animal Welfare (https://en.wikipedia.org/wiki/Universal_Declaration_on_Animal_Welfare) specifically calls for the development of appropriate welfare policies, legislation, and standards to govern the treatment and management of wildlife. Many scientists and researchers are also now arguing for the development of a compassionate or welfare-inclusive approach to conservation (4–7).

The establishment of a new discipline, “Conservation Welfare,” has been suggested to integrate the perceived competing schools of thought of conservation scientists who generally emphasize “fitness” and welfare scientists who generally emphasize “feelings” (5). There are also growing calls for the legal protection of wild animals to take account of their welfare (8, 9) and for conservation and animal protection governance systems to be better integrated (10). Certain legal frameworks such as the US Marine Mammal Protection Act mandate emergency responses to individual animals in distress, or prohibit certain forms of harassment, but fall short of taking an overview of all factors that might affect all dimensions of animal welfare.

The role of international conservation bodies is crucial when considering the welfare of free-ranging wild animals that cross national boundaries. Extensive and coordinated research effort is required to understand these animals' life histories and their likely exposure levels to human activities arising in contexts of industry, agriculture, transport, leisure, tourism, or warfare. Assessing the welfare of cetaceans (whales, dolphins, and porpoises) provides a particularly strong demonstration of this point. Tremendous multinational efforts have been expended in the past 75 years to conserve cetacean species and regulate whaling activities, including the establishment of the International Whaling Commission (IWC) in 1946. The IWC aims to conserve species and regulate whaling activities, and the experts and delegates who attend IWC meetings have amassed a wealth of information on cetacean population numbers and trends, and increasingly they seek to work alongside other scientists to address the full range of human activities that threaten cetacean populations (11). A recent comprehensive review of these threats is provided by De Vere et al. (12). The IWC has particular concerns about five categories of human activity. First, research suggests that over 300,000 whales and dolphins die annually due to ***entanglement*** in fishing gear, with others trapped in other forms of marine debris (IWC^1^). Second, the number of whales and dolphins killed by ***ship strikes*** may be high, although many collisions between larger vessels or smaller whales are likely to be unreported. The IWC has produced a strategic plan in this area (IWC^2^). Third, there is a concern relating to the role of human activities in cetacean ***strandings*** and the development of optimum response strategies (IWC^3^). Fourth, the IWC recognizes that there may be short- and long-term impacts of ***whale watching*** and wishes to promote responsible development in this area (IWC^4^). Fifth, there is a long-standing concern about the impact of ***marine contaminants*** and a degraded marine environment on cetacean population health (IWC^5^). In all of these areas, the IWC has promoted and supported active conservation research efforts and is therefore well-placed to contribute to a parallel assessment of human impacts on cetacean welfare. This is an important global goal (13). The long life span of cetaceans, as well as their late maturity and high position in the food chain, increases their susceptibility to some of these anthropogenic threats (14). In addition, the extraordinary cognitive and communication abilities of cetaceans (15, 16) and the longevity and strength of their social bonds (17, 18) jointly suggest that cetaceans possess a strong and refined sentience (19) and a capacity for suffering and enjoyment.

It has been argued that, in an ideal world, “*a large pool of independent scientists would study animal welfare issues in conservation and report on every industry and context that affects animals*” (20). This rarely occurs due to lack of awareness, lack of funding, political considerations, or difficulties in obtaining relevant animal-based data. In addition, the concept of animal welfare is complex and multifaceted. In this article, we describe how, despite these difficulties, a process for assessing the welfare of wild cetaceans was initiated in 2016 and explain how it references the Five Domains framework for animal welfare assessment (21). We discuss how we have refined this initial process and argue that a simple framework that we have developed and tested [welfare assessment tool for wild cetaceans (WATWC)] could be employed to address a range of threat scenarios. We discuss ongoing challenges and make proposals for future work.

## Development of the Welfare Assessment Tool for Wild Cetaceans

### Initial Process and Stakeholder Engagement

The IWC has for some years taken an important role in considering the welfare impacts of hunting methods, but it had not considered the impact of other human activities on the welfare of free-living cetaceans until it agreed in 2014 to establish a working group that would (in addition to providing advice to the Commission on issues related to whale-killing methods) provide advice on all aspects associated with “*ensuring good welfare of cetaceans that are hunted or otherwise impacted by human activities*” (22). This provided a stimulus for a 2-day IWC workshop supported by a grant from the UK's Department for Environment, Food and Rural Affairs (23), which took place in Kruger National Park, South Africa, in May 2016. This workshop was attended by 33 participants from 12 countries, including national authorities from IWC member countries, veterinarians, animal welfare specialists, cetacean conservation researchers (including LB, CJ, CN, MS, JV), and experts from animal welfare organizations.

The workshop explored the concept of animal welfare arising from the utilitarian arguments of Bentham and others who were primarily concerned with the question of the capacity for suffering in animals (24). As society began to accept that animals were capable of suffering, organizations such as the Royal Society for the Prevention of Cruelty to Animals (UK 1824) were founded, initially to prevent the infliction of cruelty upon animals. As thinking about animal welfare matured, the concept of animal welfare was expanded to a consideration that non-deliberate (non-cruel) human actions might also adversely affect animals. The focus of animal welfare science is now centered on animals' needs rather than the actions of people in relation to those animals. Legal developments such as the 1997 European Treaty of Amsterdam that recognized farm animals as sentient beings, capable of suffering, have also influenced the manner in which animal welfare is assessed.

The workshop recognized that the factors that influence animal welfare are multidimensional, as reflected by the Five Freedoms concept (25). Versions of the Five Freedoms now form part of most official definitions of animal welfare, e.g., World Organization for Animal Health (26) and have provided a ubiquitous guide for welfare assessment for the past 30 years. However, a revised and reconfigured version of this framework, termed the Five Domains Model (FDM) of animal welfare (21), has potential advantages in the context of the assessment of cetacean welfare. This framework was devised to facilitate systematic, structured, and coherent assessment and grading of animal welfare compromise. It incorporates four physical/functional domains (“nutrition,” “environment,” “health,” “behavior”), which focus attention on the sources of measurable sensory inputs from within and outside the body that are likely to give rise to subjective experiences. These are then accumulated into a fifth domain, inferred mental or “affective state.”

The FDM had been applied to assess welfare in other contexts, including wildlife population control [e.g., (27, 28)] but had not previously been used to assess anthropogenic threats to wild cetaceans.

The workshop explored the contexts in which there may be an ethical responsibility for cetacean welfare. For example, a reduction in the availability of food may be due to a period of natural scarcity or to the activities of humans. The negative effect on the animal will be the same regardless of the cause, but there are differences between the two cases. An assessment of animal welfare will indicate the need for an improved diet, but the ethical imperative to provide additional food differs depending on causation. Similarly, the moral imperative for humans to intervene differs if a dolphin is injured in a fight with a shark or by a ship's propeller. The workshop agreed that it is a human responsibility to reduce anthropogenic welfare threats but not a responsibility to attempt to provide welfare enhancements or to attempt to deliver positive welfare. The workshop recognized also that conservation decisions made under such circumstances will employ yet another set of principles, and intervention may be decided not on animal welfare grounds but on factors such as the endangered status of the animal or its genetic value to its population.

The workshop participants selected four of the current non-hunting threats to free-living cetacean welfare mentioned above, namely, whale watching, ship strike, entanglement in fishing gear, and marine contaminants. These are of concern to the IWC and were selected because of the potential to include welfare considerations in the development of mitigation policies in these areas. Four independent focus groups were formed, and each evaluated one threat. Each focus group made further decisions about what scenarios to consider following free discussion. There was no systematic attempt to select scenarios, and the decisions would have been influenced by the prior knowledge and experience of the participants. Stranding was not evaluated because developing policy in this area is more difficult, and work in this area is currently focused on developing appropriate emergency responses.

#### Whale Watching

The IWC has declared that “there is compelling evidence that the fitness of individual odontocetes repeatedly exposed to commercial whale-watching vessel traffic can be compromised and that this can lead to population-level effects” (29). An overview of relevant evidence is presented by Higham (30) showing that cetaceans perceive interactions with boats as a risk. Interactions with vessels frequently disrupt activity patterns (31) and, under some circumstances, can lead to shifts in residency patterns and population decline (32, 33). The whale watching focus group identified different stressors resulting from whale-watching activities within the tourism industry and tried to quantify welfare impacts associated with each of these. The group attempted to align with existing approaches to identify exposure and impacts of whale watching as developed by IWC and other organizations (e.g., (34)]. These are typically evaluated in four different scenarios: resident coastal populations; migrating groups; during feeding; and on breeding grounds. For the purpose of this exercise, a best- and worst-case scenario was developed, with the worst being a resident coastal population repeatedly exposed to stressors and with no evidence of habituation. Using this approach, the group developed a more context-specific approach and in a non-structured discussion decided to focus mainly on short-term responses when attempting to use the FDM but with some consideration of longer-term cumulative responses. Results for longer-term impacts followed a similar pattern to short-term responses, but there was less confidence in assessments of the magnitude of impacts in the longer term. Information on the long-term and cumulative impacts of whale watching on cetacean welfare was identified as a knowledge gap.

#### Ship Strike

Following free discussion, this group decided to consider four hypothetical case scenarios of cetaceans being struck by ships where the model could be applied: a large whale hit by a large vessel [sharp trauma, death in <1 h; (35)]; a small cetacean hit by a small vessel [sharp trauma, survival for many years; (36)]; a large whale hit by a small vessel (sharp trauma, decline observed 5 months after initial incident); a large whale hit by a large vessel (blunt trauma, animal brought to port on the ship's bow with a broken vertebral column). This approach enabled the group to highlight differences between welfare and conservation outcomes, noting that while lethal vessel strikes are a conservation issue for some species, non-lethal strikes or those that are not immediately lethal present a welfare concern. The group noted that each scenario involved a retrospective review of a case where the eventual outcome was known. This led to a discussion on the best point in time to make a welfare assessment. A chronic case may present healing over time; the welfare assessment could therefore differ depending on the time and stage when welfare was assessed. Lack of data on the prevalence of wounds, healing processes, and extent to which injuries are survivable all limit welfare assessment in this context.

#### Entanglement

This group decided to consider entanglement in actively used fishing gear [the cause of most entanglements; (37)] or in discarded equipment. Four hypothetical scenarios were considered: a small cetacean entangled in a net; a large whale with a minor entanglement that was shed after some time; a large whale in a severe entanglement where it was not possible to intervene; a large whale with a potentially lethal entanglement that was able to be released through intervention by trained humans (38). An acute impact was defined according to the time taken for an animal to asphyxiate when it could not reach the surface; anything that occurred over a longer period was defined as chronic. The group noted the difficulty of using the FDM to make a judgment about an individual case at the point of observation, and was uncertain how to characterize how welfare might change over time. In discussing the case of the released animal, it was noted that welfare may not improve straightaway, and indeed wounds may persist for the rest of its life. Thus, the tool could be useful in assessing the best point to intervene, contrasting short-term impact with a better outcome and improved welfare in the longer term. The workshop noted that the Global Entanglement Network utilizes an assessment tool for judging when to intervene (39), and the FDM could potentially help to develop this tool or be a useful adjunct to it.

#### Marine Contaminants

The group explored three hypothetical case scenarios involving polychlorinated biphenyls (PCBs): pilot whales in the Mediterranean with moderate exposure and killer whales in the Mediterranean with high exposure, as well as an oil spill in the Gulf of Mexico affecting a bottlenose dolphin. Discussion focused around welfare impacts of bioaccumulation on immunosuppression and reproductive suppression including increased abortions and mortality of live-born calves. It was noted that impacts on some domains were secondary or interactive, for example, once an animal becomes diseased (as a result of immunosuppression), then nutrition and behavior may be affected by lack of ability or motivation. Impacts considered in one scenario included a case of calf mortality as a result of toxicity from high levels of PCBs in milk. In this scenario, it was noted that a measure of the welfare status of a female could improve following off-load of PCB burden to her calf (which can be up to 90%) but could also decrease due to a grieving response (40, 41). For oil spills, the group considered short (period of oil spill itself) and long-term impacts and noted the importance of the research program on impacts of the 2010 Deepwater Horizon oil spill in the Gulf of Mexico. This current assessment on marine contaminants would not have been possible without the findings from that research. The scenarios explored were chosen to take best advantage of these studies.

Each category of threat was considered in detail by a focus group that developed hypothetical scenarios and applied the FDM to its cases, using a 4-point grading scale (0 = no impact, 1 = minor impact; 2 = moderate impact; 3 = severe impact) for each domain. Focus groups commented informally on their confidence in each evaluation. The groups concluded that the FDM had performed fairly well but identified challenges including the level of detail required to produce appropriate scenarios for consideration. Each group found that the FDM could be applied only where context-specific cases were considered and that it was not useful to assess very general questions. Groups identified the lack of data or knowledge in certain areas (particularly difficulties in extrapolating from single point observations of adverse events) as key constraints for the assessment of welfare of free-living cetaceans. There was no consensus on the grading scheme or how to assess welfare over time, and data on cumulative indicators of stress are scarce. A model developed by Wolfensohn et al. (42) to capture the long-term impacts of specific welfare events over the life span of the animal (in their case, laboratory primates) was considered a potentially useful way of thinking about the assessment of long-term impacts for cetaceans. There was no consensus on whether the 4-point grading scheme used for each domain was optimal or on whether the number of points should vary for data-rich and data-poor scenarios. Some groups felt that the grading scale was too coarse and that it would have been possible and beneficial to add more points to the scale, but one group felt that this would lead to false precision in scenarios where data are scarce or missing for some domains.

### Operational Principles for the Welfare Assessment Tool for Wild Cetaceans

A 1-day workshop attended by seven participants was held in London in 2018 to consider some of the challenges identified at Kruger. This workshop led to the development of the following operational principles to support the WATWC.

Any WATWC should be considered in relation to preexisting frameworks for assessing risks to animal health and welfare (43). The major steps in such risk assessment processes are (i) problem formulation [which factors have the potential to influence animal welfare in the target population and which subset of factors (hazards) have the potential to reduce animal welfare], (ii) exposure assessment (the evaluation of the strength, duration, frequency, and pattern of exposure of the hazards relevant to the exposure scenarios developed during problem formulation), (iii) consequence characterization [the assessment of the magnitude (intensity and duration) of consequences and probability of occurrence at the individual level], and (iv) risk characterization (the qualitative or quantitative integration of results from exposure assessment and consequence characterization). Seen within this risk assessment framework, the FDM clearly facilitates consequence characterization, but other approaches are needed for problem formulation and judgment of levels of exposure.

Problem formulation is a key issue to be addressed before the FDM can be applied. Problem formulation can start from a consideration of hazards (e.g., an evaluation of the consequences of changes to shipping lanes) or from a consideration of outcome measures taken on animals (e.g., measuring injuries in certain cetacean populations and evaluating strategies for reduction). From a policy perspective, and due to a lack of existing data and difficulties in collecting direct cetacean outcome measures, a hazard-based problem formulation (43) provides a better starting point. This is most easily accomplished by considering initially how a problem might affect an individual animal on a typical day.

Information should be provided to assessors on likely levels of exposure, for example, about whether a risk factor (such as a particular type of net) is present all the time or during certain periods only, and the chances that an individual animal in the vicinity would encounter the risk factor. Where data are available, they should be used, otherwise, expert opinion should be sought. If reliable estimates are available, we propose that the number of animals affected should be highlighted in any final report or recommendation. This does not form part of consequence characterization but will be useful in policy decisions.

**Figure 1:**
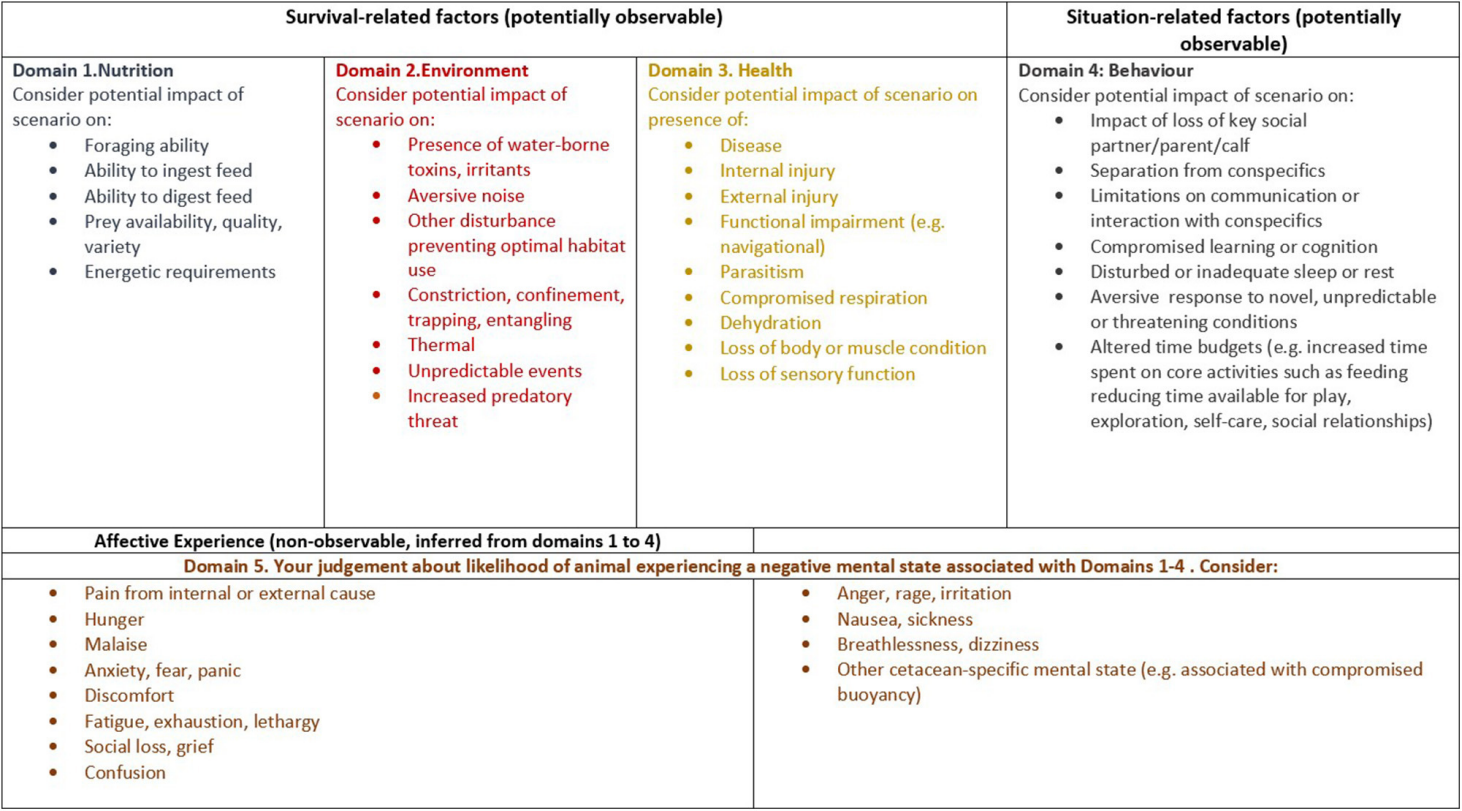
An adapted version of the Five Domains Model (44) designed to guide the assessment of the harmful effects of human activities on the welfare of wild cetaceans. Within the framework, Domains 1–4 list factors affecting cetacean welfare that could, potentially, be observable and/or measurable. Domain 5 takes aspects from each of these domains and infers the mental states that the animal may experience as a result of external stresses and challenges. These words are, necessarily, a surmised interpretation of cetaceans' mental states based on our own human emotional experiences. All negative domain states should be interpreted to mean negative states beyond an animal's normal coping capacity. It is expected that a number of the factors listed are likely to be of considerably greater significance to some cetacean species, for example, the known complexity of, and reliance on, social groupings in certain species.

The advantages of the FDM include its relative simplicity and its biological grounding. Compared with other consequence characterization frameworks (Five Freedoms or Welfare Quality®), it is less focused on principles or concerns that relate to captive animals (such as good housing or good human–animal relationships). It also differs from other frameworks by its clear separation of objective (potentially measurable) domains 1–4 and subjective (inferred “affective state”) domain 5 while showing how internal physiological and external environmental factors may generate functional affective states. For example, “*tissue injury stimulates nociceptors to propagate neural impulses to the brain where they may be transduced into the experience of pain*” (44). Because of its potential utility [it has recently and independently been suggested as a suitable framework for assessing aquatic mammal welfare; (45)], one of the outcomes of our work has been the development of a cetacean-specific version of the FDM (Figure 1). Consideration of affective state is the basis of all animal welfare assessments and yet any conscious feelings that accompany affective state cannot be directly studied. For farm or laboratory animals, judgments about affective state can be cross-checked against data about their preferences in carefully controlled experiments (46) or against studies that record subtle changes in cognition (47) or behavior [e.g., (48)] when animals are in (known) positively or negatively valenced states. Studies of this nature cannot be conducted on wild cetaceans, and so Domain 5 scoring depends on assessor knowledge of how other mammalian species (including humans) respond in analogous situations and their judgment of the extent to which wild cetaceans would be similarly affected.

The FDM itself makes no binding recommendations on specific measures, scales, confidence, and aggregation procedures for consequence characterization [although certain operational proposals were suggested by (49)]. We recommend that expert assessors should consider all of the factors that we have specified within domains (Figure 1) but should give one overall score for each domain. The approach of Beausoleil et al. (27) in asking assessors to rate their own certainty in their assessments is useful, as where uncertainty is high, it may be necessary to reframe the problem. We recommend that until further information is available about cumulative effects of threats to cetacean welfare, overall welfare risk should be considered a product of intensity × duration, with duration expressed as a proportion of the animal's expected natural life span.

## Testing the Welfare Assessment Tool for Wild Cetaceans

### Pilot Testing

Following the operational decisions, we developed six hypothetical demonstration scenarios, three concerning the ingestion of marine debris by Cuvier's beaked whales (*Ziphius cavirostris*) and three concerning the impact of tourism and whale-watching activities on the bottlenose dolphin (*Tursiops truncates)*. Each scenario was accompanied by a fact sheet that provided background information on relevant aspects of cetacean biology, health, and behavior; data about biological effects of the threats; and further reading. Areas of sparse knowledge were highlighted, and care was taken to avoid any leading comments on severity of impact or overall cetacean welfare.

Twelve animal welfare scientists were recruited as scenario assessors (eight from UK, one from Ireland, and three from Australia). They had post-doctoral experience in animal welfare science, previous experience of using the FDM in other contexts, or both. None of the assessors had specific marine mammal expertise, but the primary aim here was to pilot test the scoring framework. We developed an appropriate scoresheet that required scoring the maximum intensity (1 = least; 10 = most) of harmful impact within Domains 1 to 4 and judgment of the overall harm from the specific event affecting each individual cetacean within the Domain 5 affective state category. The assessors were asked to express confidence in their own scoring using a categorical scale; to score the duration of persistent harm following the specific event; to judge how often similar events would occur; and to assess therefore the overall proportion of life span affected by mild, moderate, or severe harm due to total events of this nature, using categorical scales provided.

All assessors returned the completed scoresheets within 2 weeks, independently and without conferring. There was relatively high variation between assessors in the scores provided, and the assessors expressed limited confidence in their own scoring. This was particularly the case for Domain 5 scores where between five and six assessors expressed low confidence around each scenario, and one assessor declined to complete the estimates of life span affected by harm for this reason. Despite this, the assessors found the pilot WATWC easy to use and appropriate in guiding their thinking. The cetacean-specific FDM was considered a useful reference. The assessors felt that reduced general background and more focused information for each scenario would be an improvement. Some minor problems with the scoring sheet and scales were identified, including a lack of clarity about when assessors should consider Domain 5 scores, a lack of a 0% option on the drop-down menu for some scoring options, and a lack of clarity about whether the life span estimates of mild–moderate and moderate–severe harm should sum to 100% (the assessors took differing approaches, with some treating these categories as independent and others as cumulative). It was also felt that there should be an increased number of categories of life span harm. The assessors liked the 10-point grading scale, which appeared to enable discrimination of impacts without leading to false precision. The feedback from this pilot testing influenced the subsequent format of case presentation and the operational details of the scoring spreadsheet used in the next phase.

### Refinement and Further Testing of the Welfare Assessment Tool for Wild Cetaceans

The pilot WATWC and results from the pilot scoring exercise were presented at a plenary session of the IWC Scientific Committee meeting, Bled, Slovenia, April 2018, and to the Whale Watching Sub-Committee at the same meeting. The committees were asked for assistance in identifying real scenarios for future welfare scoring. The Whale Watching Sub-Committee suggested the Southern Resident killer whale (*Orcinus orca*) population living off the west coast of USA and Canada, which is subject to intense whale-watching pressure, and where relevant data are available.

**Figure 2:**
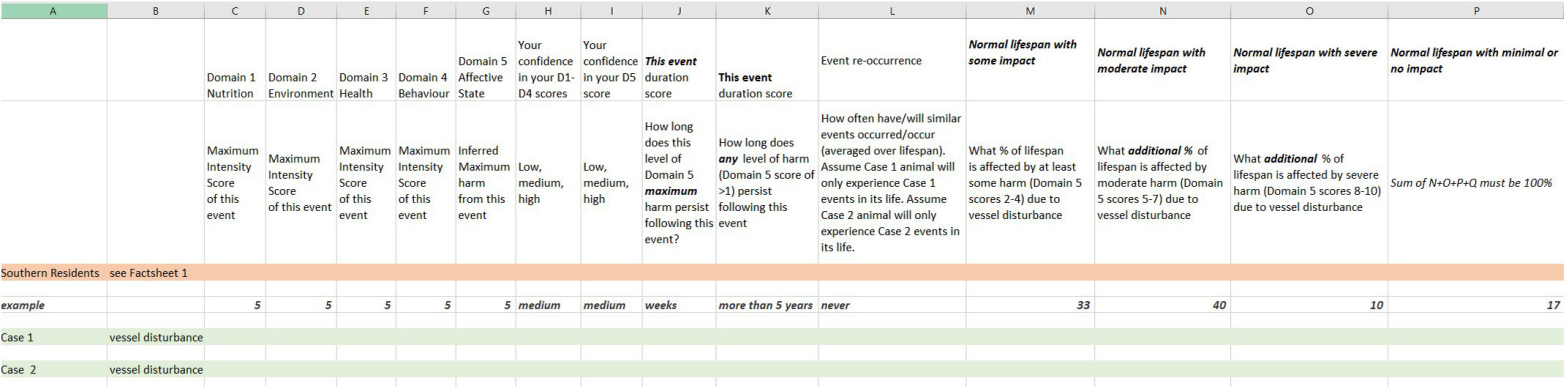
The refined version of the welfare assessment tool for wild cetaceans (WATWC) scoring sheet.

The impact of vessel traffic on Southern Resident killer whales was therefore used as a scenario to test a refined WATWC. The presentation of this scenario was guided by the feedback received during the pilot testing. A background fact sheet was prepared with accompanying references (DN, CN). Care was taken to avoid any leading or suggestive information (e.g., about affective states, welfare, or suffering) while providing pertinent scientific data on the general context, whale biology, and available data on Southern Resident killer whale behavior in the presence of vessel traffic. Two cases for scoring were drafted for a hypothetical pod X based on the data available. Based on feedback from the pilot exercise, the two cases focused on specific individual animals. The two cases (Table 1) presented different levels of exposure to vessel traffic with different levels of behavioral response observed. Thirteen potential assessors were approached, and nine agreed to participate (five welfare experts and four cetacean experts). The assessors were sent the background fact sheet (see Supplementary Material) as well as the cetacean-specific FDM and a scoresheet (Figure 2), refined in light of feedback from the pilot exercise. The functional scoresheet (in Excel format) is available upon request to interested researchers.

**Table 1:** Two cases concerning different levels of exposure to vessel traffic for individuals belonging to a hypothetical Southern Resident killer whale (SRKW) pod.

**Case 1**	**Case 2**
A SRKW 20-year-old female orca with a 2-year-old calf is a member of pod X that has been present in the inshore waters for ~50% of the time in the past year. On a day in early September, this focal female has been swimming in a subgroup with 15 companions. The other subgroups from her pod are <1 km away and swimming in a similar direction. By dusk on this day, which has been typical for this season, the subgroup has been accompanied continuously by an average of 12 vessels, with peaks of 20 vessels for a 2-h period mid-morning and a 2-h period mid-afternoon. Half of the vessels were motorized, and all remained more than 200 m from the group, most at a distance of more than 400 m. Kayaks and sail boats comprised the other observing vessels. At peak mid-morning and afternoon viewing times, background noise approached 100 dB. The focal female performed no surface active behavior in response to the vessels, but her swimming path has been more erratic during the day and she has expended 2% more energy/h during daytime periods of boat presence than during early morning or night when vessels were absent. She has remained with the subgroup all day and reunited with the rest of the pod at night.	A SRKW 20-year-old female orca with a 2-year-old calf is a member of pod X that has been present in the inshore waters for ~80% of the time in the past year. On a day in early September, this focal female has been swimming in a subgroup with 15 companions. The other subgroups from her pod are <1 km away and swimming in a similar direction. By dusk on this day, which has been typical for this season, the subgroup has been observed continuously by an average of 24 vessels, with peaks of 40 vessels for a 2-h period mid-morning and a 2-h period mid-afternoon. Three-quarters of the vessels were motorized. Of these, most remained at a distance of 200 m from the subgroup, but 10% were observed to break guidelines and approached the whales to within 90 m. Another 10% were observed idling in the path of the whales with the intention of getting a closer view as the whales approached them. At peak mid-morning and afternoon viewing times, background noise approached 140 dB. The focal female was observed performing tail slapping behavior on six occasions. On one occasion, the subgroup split into two further subgroups, and the female was briefly separated from her calf. She and her calf have become separated from the subgroup twice when following an erratic path to avoid a boat approaching directly head-on. The female has expended 5% more energy/h during the daytime periods of boat presence than during early morning or night when vessels were absent. She has remained with the subgroup for most of the day and reunited with the rest of the pod at night.

*The cases were scored using the refined welfare assessment tool for wild cetaceans (WATWC). The assessors had access to an instruction sheet, additional referenced background information (Supplementary Materials), the cetacean-specific FDM (Figure 1), and a scoresheet (Figure 2, and available on request)*.

**Figure 3:**
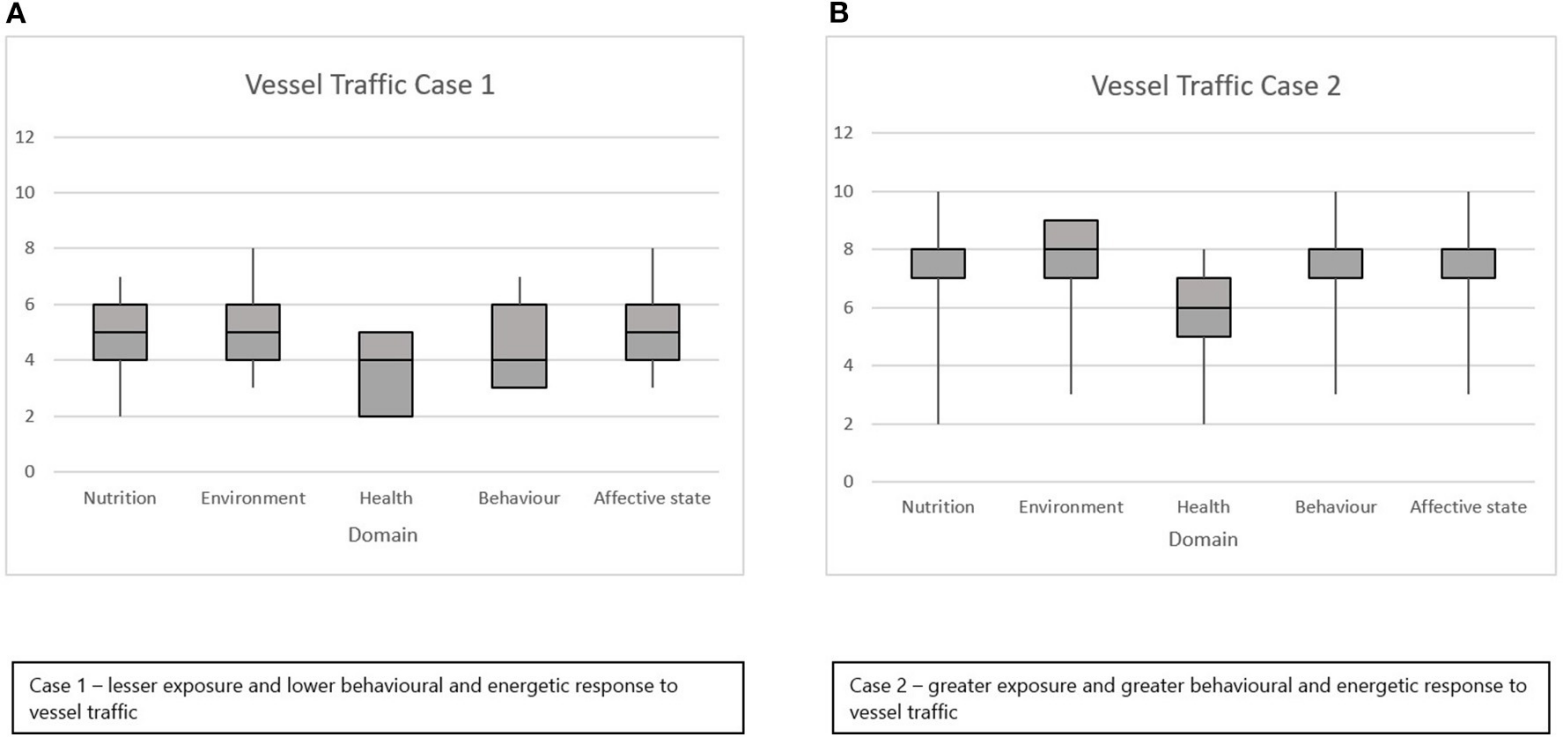
Scores for the assessed effects of lower **(A)** and higher **(B)** levels of vessel traffic and disturbance on Southern Resident killer whales using the refined version of the scoring sheet. The median scores (1 = least harm to 10 = most harm) and interquartile ranges are shown for all five domains (1 = Nutrition; 2 = Environment; 3 = Health; 4 = Behavior; 5 = Affective experience).

**Figure 4:**
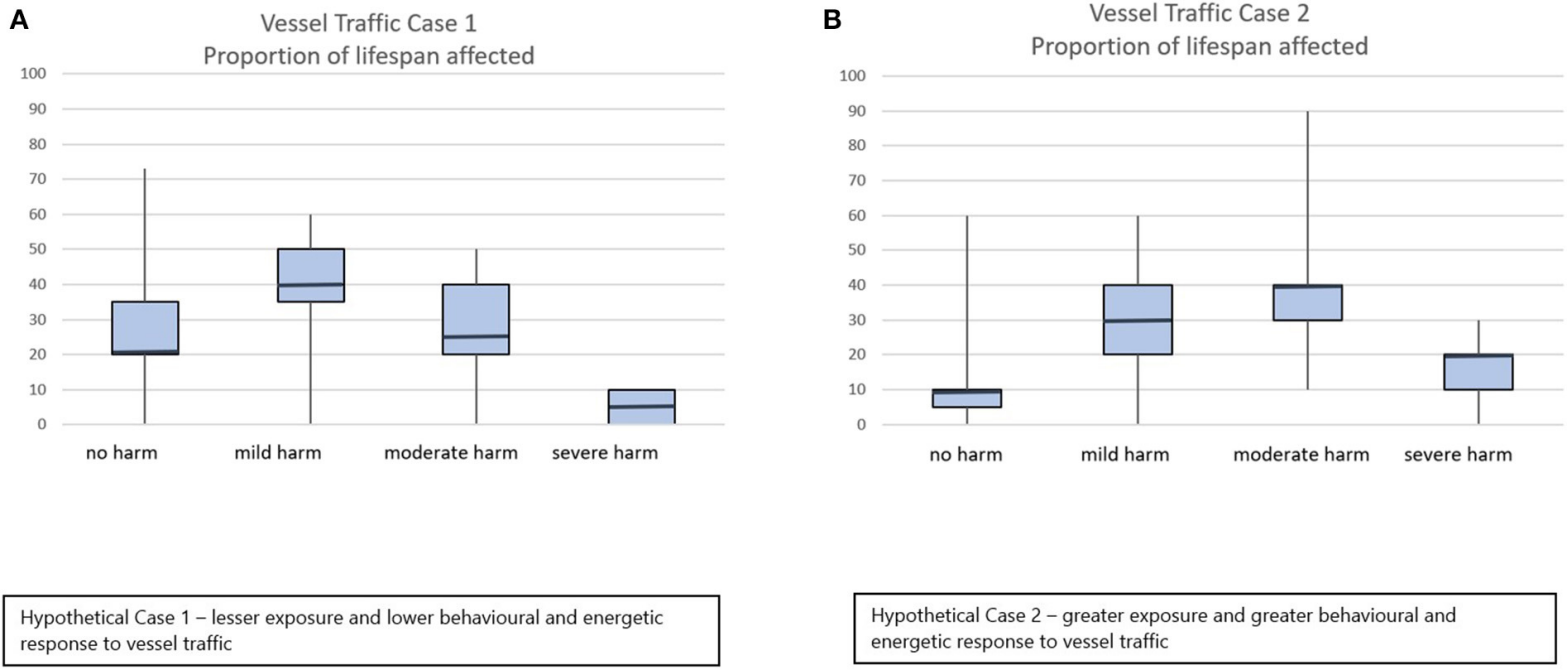
Scores for the proportion of expected life span affected by no, mild, moderate, or severe harm assessed for exposure to lower **(A)** or higher **(B)** levels of vessel traffic and disturbance on Southern Resident killer whales using the refined version of the scoring sheet. Median and interquartile ranges of judged proportions are shown.

All assessors completed the assessment independently and without conferring and returned their scores within a period of 8 weeks. The results of the severity scores for Cases 1 and 2 are shown in Figure 3. There was a clear distinction in the scores awarded for these two cases, with a median Domain 5 score of 5 for Case 1 and a median Domain 5 score of 8 for Case 2. The results of the judgment of proportion of life span affected are shown in Figure 4. Again, there was a clear differential evaluation of the two cases, with a median evaluation of 5% of life span affected by severe harm in Case 1 and 20% of life span affected by severe harm in Case 2. The majority of assessors scored very closely, with very low interquartile ranges apparent in both Figures 3, 4. However, when scoring domain severity, one assessor (not a cetacean expert) gave lower domain scores for Case 2 than the other assessors (see long downward whiskers in Figure 3B). Overall, expert background did not seem to have a major effect. Animal welfare experts gave median Domain 5 scores of 4 (Case 1) and 8 (Case 2) while cetacean experts gave median scores of 5.5 (Case 1) and 8 (Case 2). Due to the low sample size, we did not perform an extensive analysis of expert background, but this would be a useful addition to future work.

When scoring the proportion of life span affected, a different assessor (again, not a cetacean expert) considered a high proportion of life span to be unaffected by harm, being out of step with all other assessors (see long upward whisker on the “no harm” plots in Figure 4). For both cases, seven of nine assessors expressed medium confidence in their Domains 1–4 scores, eight of nine assessors expressed medium confidence in their Domain 5 scores, and one of nine expressed high confidence in his or her Domain 5 score. The individuals who expressed high or low confidence differed between cases.

Fewer questions or queries were received than during the pilot exercise, and the materials sent appeared to be largely self-explanatory. One assessor with particular expertise highlighted the need for additional specific language with respect to technical terms relating to noise levels. It was noted that noise levels should not be described as dB but as dB re 1 uPa @ 1 m RMS amplitude because measures of sounds are relative. The dB is a unit that describes sound intensity level measures relative to a fixed reference intensity and different reference intensities are used in water (1 microPascal) and air (20 microPascals) so that sound pressure levels in air are not the same as sound pressure levels in water.

Overall, the feedback received from assessors was that the WATWC had been clear and straightforward to use, but that significant challenges in assessing welfare impacts are raised by scenarios where data on cetacean health and behavioral responses are sparse.

## Discussion

We have considered and tested how the welfare of free-ranging cetaceans affected by human activities can be assessed using an expert elicitation process. Similar approaches have been used in related fields, for example, to estimate how anthropogenic disturbance from wind farm construction might affect fertility and calf survival in the North Sea harbor porpoise (*Phocoena phocoena*) (50). Asking researchers and others who are primarily involved in cetacean conservation and population management to consider how *individual* animals might be affected by human activities generated much positive discussion. We have found that a majority of stakeholders who attended the original workshop, and those subsequently engaged in this process as assessors, have regarded it a major step forward to consider individuals whether endangered or not. Our overwhelming impression is that stakeholders consider that the results from such assessments have much to add to the growing field of compassionate conservation.

Animal affective states have dimensional aspects (valence and arousal) that can be studied using preference or motivational tests (46, 51). Animal affective states could therefore be described purely in terms of their positive or negative aspects, and the strength of accompanying arousal. But to do this would overlook the fact that humans possess brains with neural structures that are shared with all other mammals (51). Discrete neural circuits underpin adaptive primal emotional responses such as fear, rage, and seeking (52) and are named as such as examples of Domain 5 states in the FDM (Figure 1). A discussion point in the workshops described here was whether more complex, cognitively regulated, and social emotions in other mammalian species should also be named. Overall, participants felt that it was helpful to provide suggestive labels for possible cetacean affective states (e.g., grief, irritation) based on similarities with ourselves in eliciting factors and observed responses. However, it was also noted that the subjective component of any cetacean affective state is beyond study, and that cetaceans may possess affective states of which we have no conception.

The scoring system for our WATWC was relatively simple, and it was greatly improved between the pilot and refined version. A higher level of assessor confidence was achieved when using the refined WATWC, although this may have arisen for multiple reasons, including more detailed case scenarios, the inclusion of real data in the background information, and the greater (average) expertise level of the assessors. Assessor confidence is not a gold standard measure of animal welfare (assessors can be wrong) but, where reliance is placed on expert judgment, it is important. In addition to high confidence, high agreement between assessors was obtained when using the refined WATWC, despite assessors scoring independently. This contrasts with problems that have arisen in other expert elicitation work with cetaceans, leading to uncertain forecasts (50). Importantly, we found that cetacean experts and welfare scientists gave very similar median domain scores and similar estimates for the highly integrated “proportion of life span affected.”

We note two areas where further consideration of operational aspects of the WATWC might be useful. First, it could be helpful to specify an acceptable level of assessor confidence. Second, it could be beneficial to give more thought to the number and independence of the factors listed within each domain (Figure 1). In developing the WATWC, we simply adapted factors from the original FDM, with the intention that they should be used as a guide to domain scoring. High domain scores were allocated if there was a high impact on any within-domain factor. If a more comprehensive list of factors is included in the future, it might be necessary to consider whether each of these would be viewed as equally important or whether any weightings should be applied.

**Figure 5:**
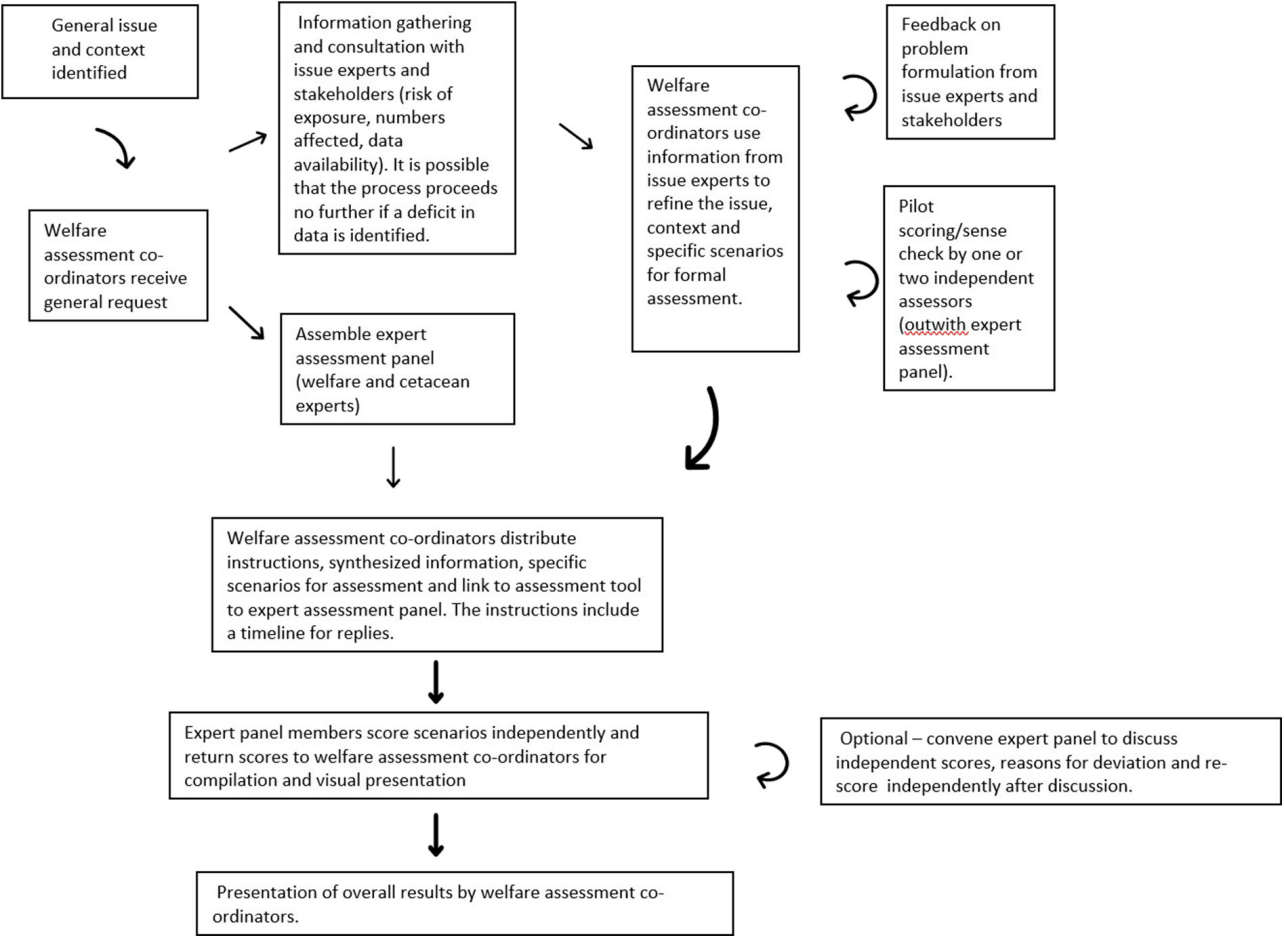
Suggested process that could be followed to implement the welfare assessment tool for wild cetaceans (WATWC) to assess new scenarios.

Our proposals to progress this work are presented in a flowchart in Figure 5. This shows the importance of engaging subject experts to assist in problem formulation and to provide data on exposure risk and context. We also recommend that both subject experts and animal welfare specialists are recruited as assessors for the expert elicitation of the impact of anthropogenic threat on the welfare of individual cetaceans. Further improvement in expert agreement can be achieved by bringing assessors together post-scoring to discuss areas of disagreement [e.g., (53)] or by use of Delphi-like processes, and this is something we advocate as part of the proposed process for developing the WATWC in the future. This is an optional step in the process represented in Figure 5. It is likely that further use of the WATWC will also lead to further refinements in process and scoring methodologies, and we encourage this ongoing improvement.

The WATWC could be used next in a variety of ways. It could be used to evaluate scenarios similar to those addressed for the Southern Resident killer whales, for example, to assess the impact of human activities on Hawaii island spinner dolphins which experience the highest exposure rates to dolphin watching in the world (54) so as to inform and influence tourist organizations to refine their activities for the benefit of whales and dolphins. Conducting assessments using the WATWC in other contexts could highlight areas where knowledge is very sparse—either information on exposure rates or numbers of animals affected or information on animal responses, thus influencing funding bodies and stimulating relevant non-invasive research in the areas of highest priority. These areas are likely to be those where severe harm is caused and a large number of animals are affected.

## Data Availability Statement

The datasets generated for this study are available on request to the corresponding author.

## Author Contributions

All authors have made a substantial, direct, and intellectual contribution to the work. LB, CJ, CN, MS, and JV participated in the Kruger workshop that initiated the process. LG, LK, CN, and MS participated in the London workshop that considered operational principles. CN, DN, and MS attended the IWC Scientific Committee meeting. CN, DN, and MS devised test scenarios and background information, and CN compiled and analyzed assessor scores. All authors participated in article preparation and writing and approved it for publication.

### Conflict of Interest

The authors declare that the research was conducted in the absence of any commercial or financial relationships that could be construed as a potential conflict of interest.

## Footnotes

^1^https://iwc.int/entanglement

^2^https://iwc.int/index.php?cID=html_191

^3^https://iwc.int/index.php?cID=html_484

^4^https://iwc.int/index.php?cID=html_66

^5^https://iwc.int/index.php?cID=html_43
