# Supplementary material for: Anthropogenic Threats to Wild Cetacean Welfare and a Tool to Inform Policy in This Area
Source: Front Vet Sci. 2020 Feb 28;7:57. doi: 10.3389/fvets.2020.00057 (PMC7058697; doi:10.3389/fvets.2020.00057)
Supplement: Supplementary file 1 [file Data_Sheet_1.pdf]

frontiers

# *Supplementary Material*

## Background Factsheet Sent to Assessors

*Permissions to use figures and data charts, and further comments on the factsheets were obtained from Ken Balcomb (Center for Whale Research) and from Taylor Shedd (Program Co-ordinator, Soundwatch of the Whale Museum, Washington).*

## General Background

### Orcas

The killer whale (*Orcinus orca*), or orca is a toothed whale of the oceanic dolphin family. Three ecotypes are recognized within the Eastern North Pacific Ocean, resident, transient and offshore orcas. Resident orcas have a fish diet and a social structure composed of matrilineal pods. Transient orcas eat marine mammals, travel in variably sized groups and have less persistent family bonds and a more ephemeral group structure than residents. Offshore orcas are typically located further from shore in relatively large groups, feeding mostly on fish, including sharks. Little is known about their social organization.

### Resident Orcas

Off the west coast of the USA and Canada, resident orcas are distributed across Northern, Southern and Alaskan communities which, despite close geographic proximity, do not interact. The focus of this welfare scoring exercise will be the well-studied Southern Resident killer whales (SRKW).

The SRKW population is organized into distinct, highly stable groups of individuals that travel together and share cultural traits including idiosyncratic calls, and hunting techniques. Within each pod, sub-pods are centered on older females. Pods may travel alongside and mix with other pods but will segregate into their original units. Females breed between approximately 10 and 40 years of life, raising a calf about once in every 5 years, and have an active family role post-menopause. Males reproduce from approximately 20 years. Both males and females participate in care of the young and calves are weaned between 1 and 2 years of age. The Northern community (approximately 200 individuals in 16 pods) comprises three clans that are distinguished by their shared acoustic traditions. Members of the same clan share many common calls. The Southern community (74 individuals in three pods) comprises a single clan, although there are detectable differences in acoustic calls types between pods.

### SRKW Geographic Range

This population consists of three pods, designated J, K, and L pods, that reside for part of the year in the inland waterways of Washington State and British Columbia (Strait of Georgia, Strait of Juan de Fuca, and Puget Sound; known collectively as the Salish Sea). The SRKW have utilized this area principally during the late spring, summer, and fall for many years although there is evidence that use of the Salish sea during the spring has reduced association with declining fish stocks (Shields et al., 2018).

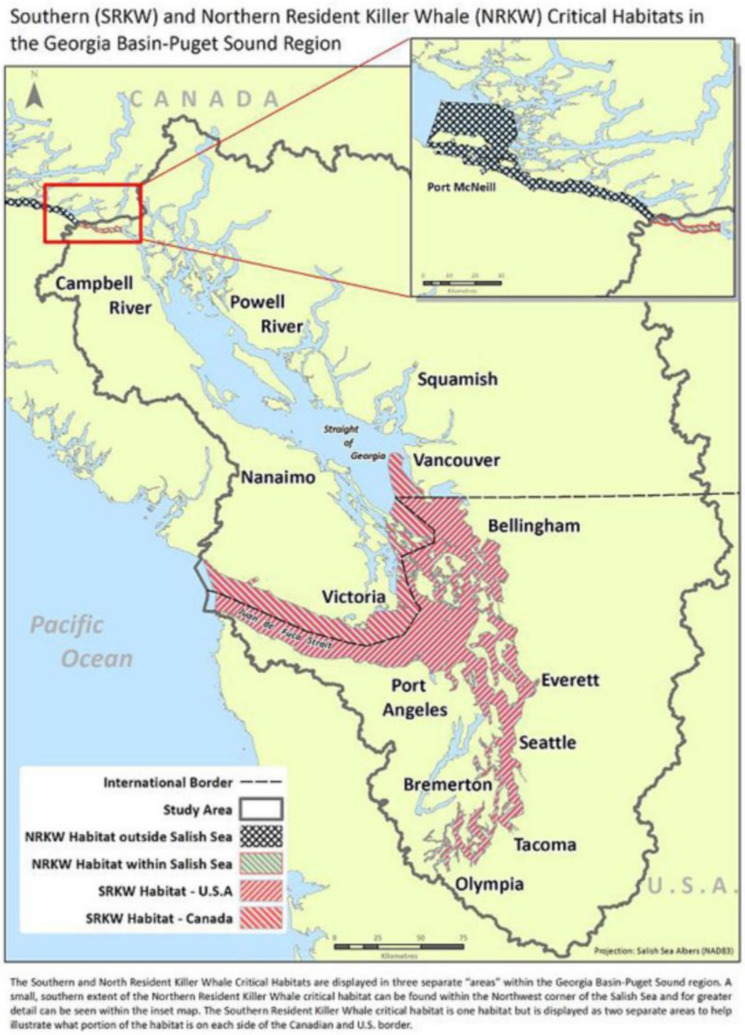


**Figure 1.** SRKW habitats, credit EPA website

https://www.epa.gov/salish-sea/southern-resident-killer-whales

During the summer, the core habitat for all pods is of the west side of San Juan Island, USA.  The three pods can be found together in this area, although there is some variability in the habitat use and distribution patterns of each pod. During the winter months pods K and L are often observed (via direct sightings, satellite tags, or passive acoustic recorders) in coastal waters of the US as far south as Monterey Bay, California, or as far north as the Chatham Strait in southeast Alaska. Pod J frequents the inshore waters during the winter months but has been observed to spend time off the coast of Washington and the southwest coast of Vancouver Island during non-summer months.

**SRKW Population Numbers and Trends**

It is likely that substantial SRKW population declines occurred co-incidentally with the rise of industrial fishing (late 1800s), and dam construction in the 1930s to 1960s. Figure 2, reproduced from the US EPA website (https://www.epa.gov/salish-sea. southern-resident-killer-whales) shows a further major decline in SRKW numbers in the late 1960s and early 1970s when many individuals were captured and shipped to marine

parks.  The source of the modelled data in this Figure (shown in light blue) is not clear and it is possible that the declines were greater than portrayed here. The dark blue data arise from accurate and reliable surveys conducted from 1976 by the Center for Whale Research.  These show that the population increased to the mid-1990s, but it has been falling again in recent years and was listed as endangered in 2005. In September 2018 there were just 74 recorded individuals.

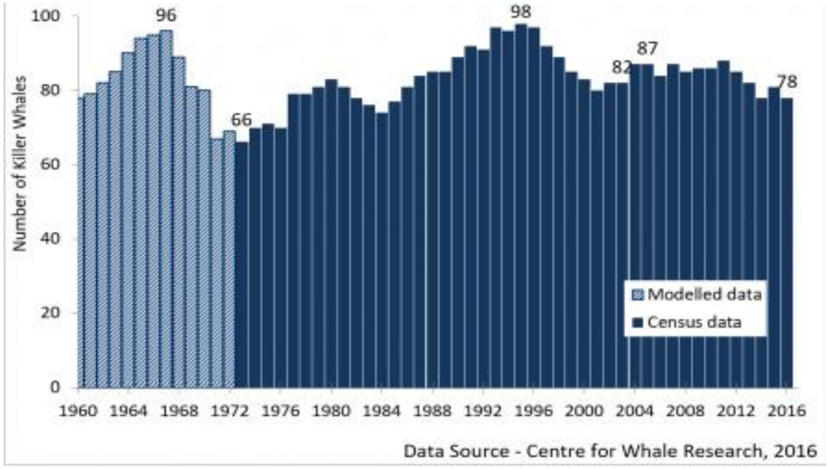


**Figure 2.**

In contrast, populations of transient and offshore orcas have increased over the same time period. Transient and offshore orcas may visit the inshore waters, but they are, on average, less exposed to vessel traffic.  Northern resident killer whales have also increased in numbers since 2002, with an annual growth rate of 2.9% and a population of 309 individuals in 2017.

**SRKW Diet**

The diet of the SRKW has been assessed by direct observation, quantitative fecal prey DNA and biopsy isotope data. Many studies, and all methods, confirm that the Chinook salmon provide the major dietary component for this population during the summer months (see Figure 3 for example data). The ingestion of Coho salmon is important later in the year, though there is high inter-annual variation in the proportion of Coho in the late season diet.

| Season | Year | Halibut | Herring | Chinook | Chum | Coho | Sockeye |
|---|---|---|---|---|---|---|---|
| Early | 2006 | 0.000 | 0.000 | 0.981 | 0.009 | 0.005 | 0.000 |
| Early | 2007 | 0.000 | 0.000 | 0.990 | 0.003 | 0.003 | 0.000 |
| Early | 2008 | 0.000 | 0.000 | 0.961 | 0.001 | 0.000 | 0.020 |
| Early | 2010 | 0.000 | 0.000 | 0.999 | 0.001 | 0.000 | 0.000 |
| Early | 2011 | 0.000 | 0.000 | 0.969 | 0.001 | 0.001 | 0.000 |
| Middle | 2006 | 0.003 | 0.000 | 0.995 | 0.001 | 0.001 | 0.000 |
| Middle | 2008 | 0.005 | 0.000 | 0.873 | 0.001 | 0.000 | 0.121 |
| Middle | 2010 | 0.005 | 0.000 | 0.971 | 0.003 | 0.020 | 0.000 |
| Middle | 2011 | 0.001 | 0.094 | 0.721 | 0.000 | 0.001 | 0.183 |
| Late | 2006 | 0.000 | 0.000 | 0.297 | 0.001 | 0.607 | 0.000 |
| Late | 2007 | 0.064 | 0.000 | 0.756 | 0.004 | 0.172 | 0.001 |
| Late | 2010 | 0.000 | 0.002 | 0.532 | 0.027 | 0.438 | 0.000 |
| Late | 2011 | 0.001 | 0.000 | 0.475 | 0.001 | 0.522 | 0.001 |
| Total | | 0.006 | 0.008 | 0.795 | 0.004 | 0.150 | 0.025 |

**Figure 3.** Proportion of prey DNA sequences from SRKW fecal samples (reproduced from Ford et al., 2016, PLOS One, under Creative Commons Attribution license)

Contaminant data from blubber samples, which integrate information over a longer time period, confirm that pods K and L forage in coastal waters off California and ingest more DDTs.

**SRKW Impact of Vessel Traffic**

The focus of this welfare scoring exercise will be the direct impact of vessel traffic on representative individuals of the SRKW population, during the peak summer months. Indirect effects due to increased pollution levels will not be considered in this exercise. High vessel traffic has been highlighted as contributing factor to the SRKW population decline. In 2011, federal regulations were implemented to prohibit vessels approaching within 183m of whales, and to prohibit stopping or parking in their swimming path.

Vessels include commercial whale-watching boats (rigid hull inflatables, passenger, and cruiser style) which are active from 09:00 to 21:00 in the summer, and which comprise approx. 45% of vessels near SRKW (Seely et al., 2017). Also engaged in whale watching activities are private recreational vessels (27% of vessels near whales) and increasing numbers of canoes and kayaks in recent years (7% of vessels near whales). Research, military, sport and professional fishing vessels, ferries, cruise ships and intermittent freight vessels also occur in proximity with SRKW locations.

***Exposure – Whale Watching Activities***

The most accurate annual reports on vessel numbers in the region are produced by Soundwatch, an organization founded by The Whale Museum in 1993, and funded to prevent vessel disturbance in the Salish sea.

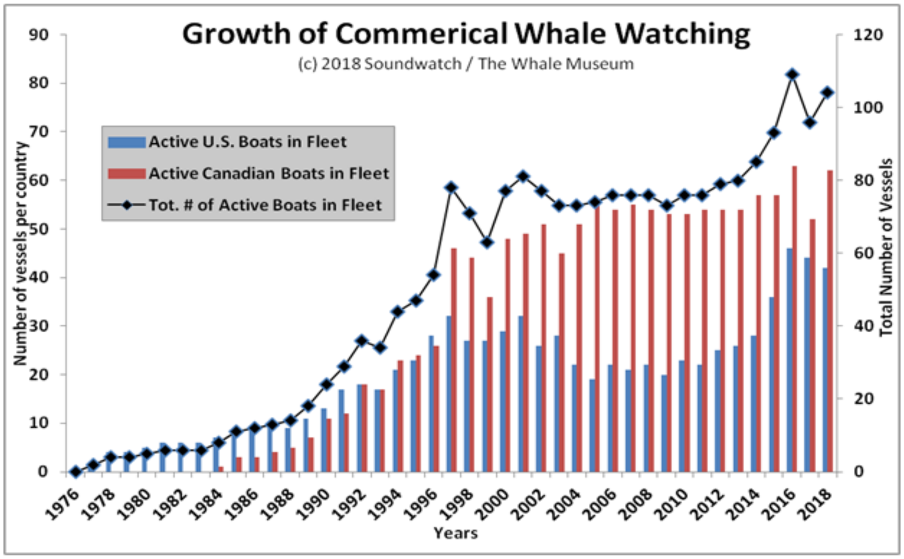


**Figure 4**. shows that the fleet number was relatively stable at around 75 vessels between 2005 and 2015 before increasing in 2016 then dropping back in 2017. It is too early to know whether this reduction will be sustained. Credit Taylor Shedd- Program Coordinator, Soundwatch of The Whale Museum, Friday Harbor, Washington USA.

Between 1998 and 2006 SRKWs consistently had an average of nearly 20 vessels within a half-mile (approx. 800 m) of their location between 09:00 and 18:00 from May through September. Maximum boat counts during this period ranged from 69 to 120 vessels (Fig 6). The average number of boats close to SRKWs has declined since 2006, to an average of 10 in 2018.

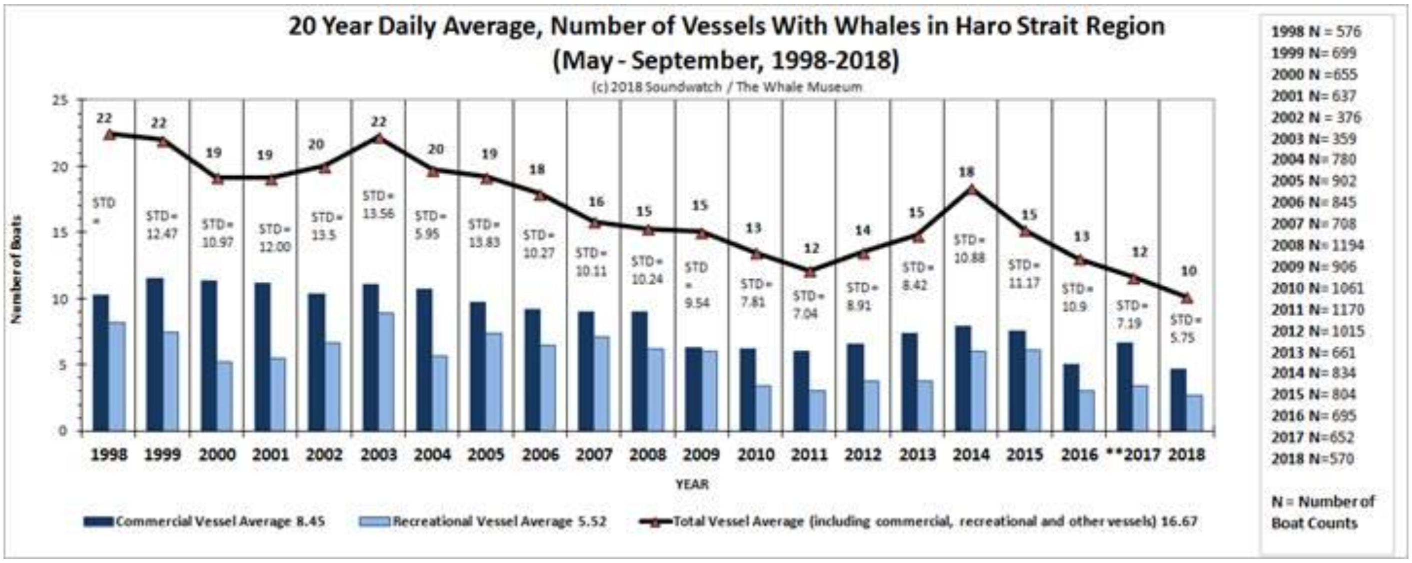


**Figure 5.** shows the number of vessels within approximately 800 m of SRKWs, credit Taylor Shedd- Program Coordinator, Soundwatch of The Whale Museum, Friday Harbor, Washington USA.

Seely et al. (2017) ascribe this decline to improved compliance with 2011 regulations, although those regulations only specifically prohibit approach within 183 m. It is important to consider that these apparent declines may partly be because of pods splitting into smaller sub-pods, also dividing the boat traffic. Other

regulations and guidelines are often contravened, with monitors recording many incidents (approx. 3 to 5 per hour) where boats approach within 200 or 100 m of whales, cross or stop in the whale's swimming path (Seely et al., 2017).

***Exposure – Shipping***

At least 20 ships make their way through the Haro Strait, on the west side of San Juan Island in the Salish Sea. This is a critical core summer use area (Hauser et al. 2007) and one where there are key foraging locations that have been utilized for many decades, although the proportion of time spent foraging in these locations is declining (Noren and Hauser, 2016). Veirs et al. (2016) combined hydrophone measurements with vessel location data and found elevations (20 to 30 dB for low frequencies, and 5 to 13 dB for high frequencies [1]). The elevation in noise level at high frequency may interfere with orca communication and echolocation.

***Effects on Behaviour***

Many studies have demonstrated that vessel presence affects the movement behaviour of orcas (e.g. Jelinski et al., 2002). Specific studies of SRKWs have studied the effect of vessels at distances within 1000, 400 and 100 m of whales (Lusseau et al., 2009). There was no effect of boat presence on total number of behavioral transitions, but boats within 400 m or (more so) within 100 m, decreased the likelihood that a whale would continue to forage. In addition, when boats were within 100 m, the overall proportion of time spent foraging declined from approx. 0.7 to 0.6, with a concomitant increase in proportion of time spent travelling. The reactions of the three pods to boats were not significantly different.

A study by Williams et al., (2009) found significant, but small (only modest levels of variation explained) and non-linear effects of boat presence and proximity on SRKWs. For example, dive times increased as boat numbers increased to approx. eight, but then declined as boat numbers increased further. Swimming speed increased when approx. eight boats were within 400 m but tapered when more than 12 boats were within this distance. Swimming paths were more erratic when more boats were in view. Some non-linear effects may be explained by whales adopting different strategies according to boat number and proximity. Avoidance responses to a few boats (e.g. adopting an erratic path) may not be effective when many boats are present. Surface active behaviors including fin and tail slaps, breaches, cartwheels and spy-hops (orca in vertical position, with head above water, maintaining position with tail fluke) occur frequently when vessels are within 200 m of whales, particularly around the time of closest approach. These potentially energetically costly behaviors thus appear to be a response to close approach (Noren et al. 2009).

***Effects of Noise***

Noise from vessels falls within the orca hearing range of approx. 0 to 100 kHz. Noise may mask echolocation signals, reducing ability to locate fish (Erbe, 2002). Holt (2008) estimated an 88% reduction in echolocation efficiency with a motorized vessel within 91 m. Motorized vessels affect the spacing of orcas more than sailboats or kayaks (Jelinski et al., 2002).

Foote et al., (2004) found no significant difference in primary calls or call rate of SRKWs, in the presence or absence of boats in 1977 to 11981; 1989 to 1992, but a significant increase of approx. 15% in call duration for all three pods in 2001 to 2003, when average numbers of boats were much greater. Increases in call duration were also reported by Wieland et al. (2010) for 16 out of 21 calls.

Holt et al., (2008) found that background noise level was clearly correlated with vessel number, and that as background noise increased (from approx. 100 dB to 120 dB), SRKW increased their call amplitude by 1 dB for every 1 dB increase in background noise (from approx. 145 dB to 165 dB). This compensatory Lombard effect, occurred for each of 7 specific call types (Holt et al., 2011).

***Collisions***

Occasional collisions have been recorded between boats and Resident orcas in British Columbia. Two fairly recent deaths of Southern Resident killer whales were definitely caused by impacts with ships. One 6.5-year-old male (L98), who was (unusually) solitary and boat-interactive died when he collided with the propeller of a tugboat in 2006. In December 2016, an 18-year-old male (J34) was found dead in waters off British Columbia. Necropsy results revealed that the cause of death was trauma that was likely caused by impact with a ship. In 2012, a 3-year-old female (L112) that died from trauma from an unknown source was found stranded on the coast of Washington (Noren, personal communication).

***Energy Expenditure***

There are concerns that the diminishing availability of Chinook salmon may be becoming a limiting factor in the breeding success of the SRKW population. Reduced foraging behaviour may reduce energy intake (estimated up to 18% reduction in presence of vessels compared with absences, Williams et al., 2006). Increased effort expended in communication and altered behavioral strategies may increase energy loss (Bain, 2002; Williams et al., 2006). Although the cumulative cost of these changes in behaviour may be relatively small, the impact of switching from foraging to travelling may have large effects on overall energy balance. Lactating females and growing calves may be particularly susceptible to reduced energy intake.
